# Antiviral Activity of the Propylamylatin^TM^ Formula against the Novel Coronavirus SARS-CoV-2 In Vitro Using Direct Injection and Gas Assays in Virus Suspensions

**DOI:** 10.3390/v13030415

**Published:** 2021-03-05

**Authors:** Ashley N. Brown, Gary Strobel, Kaley C. Hanrahan, Joe Sears

**Affiliations:** 1 Institute for Therapeutic Innovation, Department of Medicine, College of Medicine, University of Florida, Orlando, FL 32827, USA; Kaley.Hanrahan@medicine.ufl.edu; 2 Department of Plant Sciences, Montana State University, Bozeman, MT 59715, USA; uplgs@montana.edu; 3 Center for Lab Services/RJ Lee Group, Pasco, WA 99301, USA; JSears@rjleegroup.com

**Keywords:** SARS-CoV-2, COVID-19, antiviral agent, *Muscodor albus*, volatile organic compounds

## Abstract

Severe acute respiratory syndrome coronavirus 2 (SARS-CoV-2), the causative agent of novel coronavirus disease 2019 (COVID-19), has become a severe threat to global public health. There are currently no antiviral therapies approved for the treatment or prevention of mild to moderate COVID-19 as remdesivir is only approved for severe COVID-19 cases. Here, we evaluated the antiviral potential of a Propylamylatin formula, which is a mixture of propionic acid and isoamyl hexanoates. The Propylamylatin formula was investigated in gaseous and liquid phases against 1 mL viral suspensions containing 10^5^ PFU of SARS-CoV-2. Viral suspensions were sampled at various times post-exposure and infectious virus was quantified by plaque assay on Vero E6 cells. Propylamylatin formula vapors were effective at inactivating infectious SARS-CoV-2 to undetectable levels at room temperature and body temperature, but the decline in virus was substantially faster at the higher temperature (15 min versus 24 h). The direct injection of liquid Propylamylatin formula into viral suspensions also completely inactivated SARS-CoV-2 and the rapidity of inactivation occurred in an exposure dependent manner. The overall volume that resulted in 90% viral inactivation over the course of the direct injection experiment (EC_90_) was 4.28 µls. Further investigation revealed that the majority of the antiviral effect was attributed to the propionic acid which yielded an overall EC_90_ value of 11.50 µls whereas the isoamyl hexanoates provided at most a 10-fold reduction in infectious virus. The combination of propionic acid and isoamyl hexanoates was much more potent than the individual components alone, suggesting synergy between these components. These findings illustrate the therapeutic promise of the Propylamylatin formula as a potential treatment strategy for COVID-19 and future studies are warranted.

## 1. Introduction

*Muscodor albus* is a novel endophytic fungus, isolated from *Cinnamomum zeylanicum,* that produces over two dozen volatile organic compounds (VOCs) that demonstrate potent antimicrobial activity against a wide range of both human and plant pathogens [1,2]. These VOCs were characterized using gas chromatography/mass spectrometry (GC/MS) to determine the identities and amounts of each individual compound. It was established that the small molecular weight organic acid, isobutyric acid, was central to the bioactivity of the VOCs [2,3]. However, when isobutyric acid was placed in combination with one or more complex esters that had little or no activity alone (depending on the target organism), antimicrobial activity was substantially increased and resulted in a synergistic inhibitory response [4]. Of note, when isobutyric acid was combined in the laboratory with the esters in the same relative molar ratio that they were measured via GC/MS, the “artificial” mixture virtually mimicked the activity of the fungus itself [2,3]. This antimicrobial activity was predominantly characterized utilizing the *M. albus* compounds in the volatile state [1,2].

Subsequently, for utility and intellectual property purposes, and after extensive testing, it was learned that propionic acid could be substituted for isobutyric acid and isoamyl hexanoates (two esters of the 2 and 3 isomers of the alcohol) could serve as a substitute for other esters, aldehydes and alcohols in the *M. albus* mixture without compromising biological activity. Interestingly, neither the propionic acid nor the isoamyl hexanoates have ever been noted as products of *Muscodor* sp. In the gas phase as well as in MIC (minimum inhibitory concentration) tests, the propionic acid/isoamyl hexanoate mixture, now named Propylamylatin formula, was biologically active against a wide range of both human and plant pathogenic fungi and bacteria, including such microbes as *Listeria* sp., *Salmonella* sp., drug-resistant *Staphyloccus aureus*, *Clostridium* sp., *Xanthomonas* sp. *Candida albicans*, *Pythium ultimum, Rhizoctonia solani,* and many others [5]. The Propylamylatin formula also targeted many other bacteria and fungi that are relatively resistant to antimicrobial interventions, including *Trichoderma* sp and *Lactobacillus* sp. [5]. Unfortunately, the exact mode of action of the formula is unknown, but it has been suggested that one or more physio-chemical mechanisms involving membrane proteins might be involved.

Despite the well-documented antibacterial and antifungal properties of the Propylamylatin formula, antiviral activity had yet to be noted [5]; that is, until the 2013 to 2014 outbreak of porcine epidemic diarrhea (PED) caused by the porcine epidemic diseases virus (PEDv) in the United States. PED spread quickly throughout the pig population; one grower in Montana lost 850 piglets to PEDv infection within weeks of the disease reaching his locale. The Propylamylatin formula solution containing the propionic acid/isoamyl hexanoate electrolyte mixture was administered to tens of animals suffering with PED to assist in rehydration and electrolyte balance. Many of the animals that received the solution improved rapidly, showing evidence of decreased clinical signs after 5 h post-administration, and all animals completely recovered by 24 h (Strobel, unpublished record of Ecoplanet Environmental L.L.C). Interestingly, PEDv is a coronavirus and this fact led us to investigate the distinct possibility that the Propylamylatin formula may be helpful in other coronavirus infections [6].

Thus, the immediate purpose of this report is to show, via gas and direct injection assays with virus suspensions, that the Propylamylatin formula is active against SARS-CoV-2.

## 2. Materials and Methods

### 2.1. Cells, Viruses, and the Propylamylatin Formula

Vero E6 cells were purchased from the American Type Culture Collection (ATCC; Manassas, VA, USA) and cultured in Minimum Essential Medium (MEM; Corning Cellgro, Tewksbury, MA, USA) supplemented with 10% fetal bovine serum (FBS; Hyclone, Logan City, UT, USA) and 1% penicillin-streptomycin solution (Hyclone). Cells were maintained at 37 °C, 5% CO_2_ and sub-cultured twice weekly to ensure sub-confluency.

The USA-WA1/2020 strain of SARS-CoV-2 was deposited by the Centers for Disease Control and Prevention and obtained through BEI Resources, NIAID, NIH (NR-52281). Viral stocks were propagated in Vero E6 cells for two days. Supernatants were collected, clarified by high-speed centrifugation, aliquoted and frozen at −80 °C. Viral stock titers were determined by plaque assay on Vero E6 cells. All work with SARS-CoV-2 was conducted under Biosafety Level 3 plus conditions at the Institute for Therapeutic Innovation (University of Florida, Orlando, FL, USA) using safety protocols approved by the University of Florida Institutional Biosafety Committee (BIO5525 approved 11 March 2020).

The Propylamylatin formula consists of a mixture of propionic acid and isoamyl hexanoates at a ratio of 7:2 (*v/v*) or with a molar ratio of 10.2:1. The isoamyl hexanoates are present in the mixture of esters at a ratio of 99:1 of the 3 to the 2 isomer. These compounds were obtained from Eccelentia International Co. (Fairfield, NJ, USA). Other chemicals were obtained from Sigma Aldrich (St. Louis, MO, USA).

### 2.2. The Propylamylatin Formula Gaseous and Direct Injection Evaluations against SARS-CoV-2

**Figure 1:**
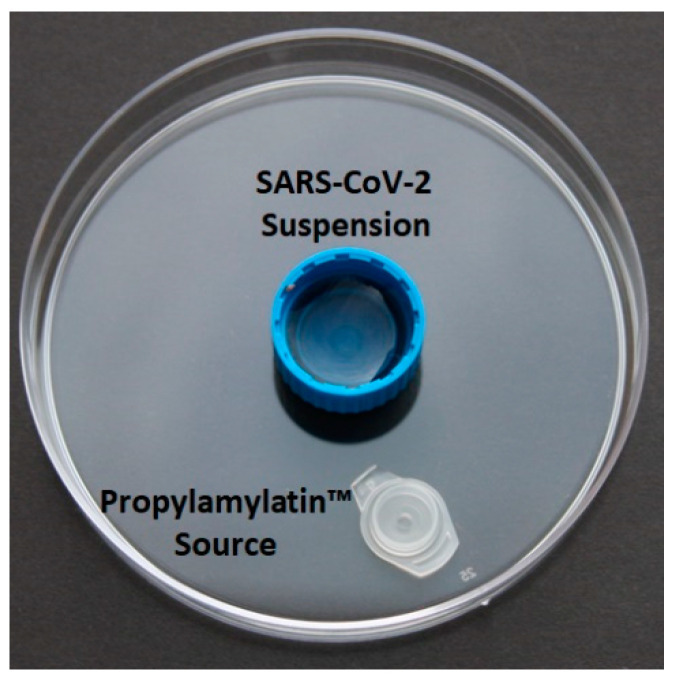
Layout for the vapor evaluations of the Propylamylatin formula against SARS-CoV-2. The blue plastic cap in the center holds 1 mL of SARS-CoV-2 diluted to 10^5^ PFU/mL in MEM. The white cap holds 100 µls of the Propylamylatin formula or PBS and is placed 2.5 cm away from the center cap. The petri dish was rimmed with parafilm to prevent gas from escaping the environment. Two experimental plates and two control plates were harvested at each time point.

For vapor (gas phase) evaluations of the Propylamylatin formula, SARS-CoV-2 was diluted to a concentration equivalent to 10^5^ plaque forming units (PFU)/mL in MEM. Aliquots containing 1 mL of the virus suspension (10^5^ PFU) were placed into plastic caps and secured to the surface of 100 mm × 15 mm polystyrene petri dishes with double sided tape directly in the middle of the petri dishes. Small plastic caps (the lid from a standard microcentrifuge tube) containing 100 µls of the Propylamylatin formula were placed 2.5 cm away from the virus-containing caps. The top of the petri dish was sealed with parafilm and plates were incubated at both room temperature (~18 °C) and 37 °C for various lengths of time (Figure 1). Since the removal of the top cover of the petri dish immediately destroys the gas atmosphere of that dish, one petri dish for each experimental arm was harvested at each time point. Time-matched control plates were included in the experimental design and set up exactly as described above with the exception that 100 µls of PBS was placed inside the white small plastic cap. At the time of sampling, viral supernatant was transferred from the plastic cap into a microfuge tube and frozen at −80 °C until the end of the study. The viral burden was quantified by plaque assay on Vero E6 cells for all samples simultaneously. Each experimental arm was assayed in duplicate to determine between sample variability and at least two independent assays were conducted on different days to address between-day variability.

Additional vapor (gas phase) evaluations were conducted with various volumes of the Propylamylatin formula against SARS-CoV-2 at 37 °C. Studies were conducted as described above and shown in Figure 1, but for these experiments different volumes ranging from 25 µls to 100 µls of the Propylamylatin formula were placed in the white small plastic cap. Time- and volume-matched PBS controls were included in this experiment. All experimental arms were sampled in duplicate and processed as described above. The viral burden was determined by plaque assay on Vero E6 cells and two independent studies were conducted on two different days.

The ability of the Propylamylatin formula to inactivate SARS-CoV-2 when directly injected into a viral suspension was also evaluated. For these studies, various volumes of the Propylamylatin formula were directly inoculated into 1 mL aliquots of SARS-CoV-2 diluted to a concentration of 10^5^ PFU/mL in MEM and incubated at 37 °C. At various times post-inoculation, aliquots from each experimental arm were harvested, in duplicate, and frozen at −80 °C until the end of the study. The viral burden was quantified by plaque assay on Vero E6 cells for all samples simultaneously. At least two independent studies were conducted on different days to address between-day variability.

The anti-SARS-CoV-2 activity of the individual components making up the Propylamylatin formula (i.e., propionic acid alone and esters alone) was evaluated in the amounts in which they are present in the formula at the 7:2 (*v/v*) ratio, using the direct injection methods described above. Isobutyric acid (the original acid produced by *M. albus*) was substituted for the propionic acid in the mixture and also assessed via direct injection.

### 2.3. SARS-CoV-2 Plaque Assay

Vero E6 cells were seeded into six-well plates and incubated until confluency at 37 °C, 5% CO_2_. Viral samples were thawed on ice, serially diluted 10-fold in MEM, and 100 µls of each dilution was inoculated onto Vero E6 monolayers. Plates were incubated for 1 h to allow for viral attachment and were shaken every 15 min to maintain an even distribution of the viral inoculum on the cell monolayer. Monolayers were then overlaid with MEM containing a final concentration of 0.5% agar (*w/v*) and 5% FBS (*v/v*). Two days later, a secondary overlay containing MEM with a final concentration of 0.5% agar, 1% FBS, and 0.007% neutral red solution was added to each well of the six-well plate. Plaques were counted on the following day (72 h post-infection) with the naked eye.

### 2.4. Gas Chromatography/Mass Spectroscopy (GC/MS) and Gas Chromatography/Flame Ionization Detection (GC/FID) Plus Colorimetric Titration of the Virus Wells

We estimated the amount of dissolved propionic acid and isoamyl hexanoates in MEM when the Propylamylatin formula was administered via gas phase, and the resulting concentrations served as a guide for the design of the direct injection experiments. For these assays, petri dishes were set up exactly as described above for the Propylamylatin formula gas phase evaluations with the exception that the virus was not included in these studies. Propionic acid was quantified using two different methods. The first method was via titration of the MEM (which contains phenol red, a pH indicator) with a 1 M NaOH solution. The second method was by GC/FID in which samples, taken from the wells, were diluted 20-fold, separated by GC, and the amounts measured by FID. The samples were injected into a Hewlett Packard 6890 gas chromatograph containing a 30 m × 0.25 mm inner diameter ZB Wax capillary column with a film thickness of 0.50 mm. A thermal program of 30 °C for 2 min followed by an increase to 220 °C at 5 °C min^−1^ was applied. Ultrahigh-purity helium gas was used as the carrier gas and the initial column head pressure was 50 KPa. The quantification for both methods was conducted in triplicate.

The presence of the esters was determined both qualitatively and quantitatively by GC/MS using a Hewlett Packard 6890 gas chromatograph. The samples were diluted two-fold and the initial identification of the esters found in the analysis was made via library comparison using the National Institute of Standards and Technology (NIST) database. For GC/MS analyses, authentic samples of isoamyl hexanoates were used to generate standard curves to interpolate the concentrations of each compound in the media samples.

### 2.5. pH Determination of Virus Diluent Media

Various volumes of the Propylamylatin formula were added to 1 mL of MEM. The pH of the media was measured using MColorpHast^™^ pH test strips and indicator papers (MilliporeSigma, Burlington, MA, USA) at various times ranging from 15 min to 24 h post-Propylamylatin-formula addition.

### 2.6. Statistical Analyses

The volume of the Propylamylatin formula that reduces viral burden by 90% (EC_90_) was determined on individual time points as well as over the entire duration of the experiment. For analyses at individual time points, an inhibitory Sigmoid-Emax model (Hill model) was fit to the viral burden data from each experimental arm generated at that time point. Analyses of the entire data set was determined by first calculating the area under the viral burden time curve (AUC_VB-Time_) for each experimental condition. An inhibitory sigmoid-Emax model (Hill model) was then fit to the AUC_VB-time_ values. All inhibitory sigmoid-Emax models were fit to the data using GraphPad Prism software (version 7.02).

## 3. Results

### 3.1. Antiviral Activity of the Propylamylatin Formulavapors against SARS-CoV-2

**Figure 2:**
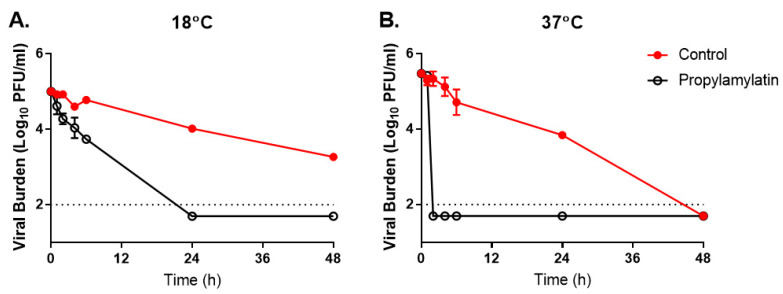
The effect of the Propylamylatin formula vapors on the infectivity of SARS-CoV-2. A suspension of 10^5^ PFU of SARS-CoV-2 was exposed to the Propylamylatin formula vapors resulting from a 100 µL aliquot of the Propylamylatin formula and incubated at either (**A**) room temperature (18 °C) or (**B**) body temperature (37 °C). At various times post-Propylamylatin-formula exposure, viral suspensions were harvested, and viral burden was quantified by plaque assay in Vero E6 cells. Viral burden is reported as log_10_ PFU/mL. Data points correspond to the geometric mean of two independent samples and error bars represent one standard deviation. The horizontal dashed line signifies the plaque assay limit of detection.

We first evaluated the ability of Propylamylatin formula vapors to inactivate infectious SARS-CoV-2 at room temperature (~18 °C) and body temperature (37 °C). SARS-CoV-2 naturally loses infectivity over time, as the viral burden decreased in the control arms at both temperature conditions (Figure 2). The viral stability, however, was heavily influenced by temperature. At 18 °C, viral burden decreased by 1-log_10_ PFU/mL at 24 h and by 1.7-log_10_ PFU/mL at 48 h in the control arm (Figure 2A). In contrast, control titers were reduced by 1.7-log_10_ PFU/mL at 24 h and were undetectable at 37 °C after 48 h (Figure 2B). These findings indicate that cooler temperatures allow for a longer period of infectivity for SARS-CoV-2.

Propylamylatin formula vapors were effective at neutralizing infectious SARS-CoV-2 and exposure to these vapors resulted in viral burden levels below the assay limit of detection for both temperature conditions at time points that were markedly faster than those observed for the corresponding controls (Figure 2). The duration of the Propylamylatin formula exposure to achieve complete viral inactivation (where titers were below the limit of detection) was heavily influenced by temperature. Inactivation of SARS-CoV-2 was markedly more rapid at 37 °C, as infectious virus was undetectable after only 2 h of exposure to the Propylamylatin formula vapors (Figure 2B). The reduction in viral burden was more gradual at 18 °C and 24 h of exposure time was required to completely inactivate infectious virus at room temperature (Figure 2A). These findings demonstrate that the Propylamylatin formula vapors are effective at neutralizing infectious SARS-CoV-2 and neutralization is faster at higher temperatures.

**Figure 3:**
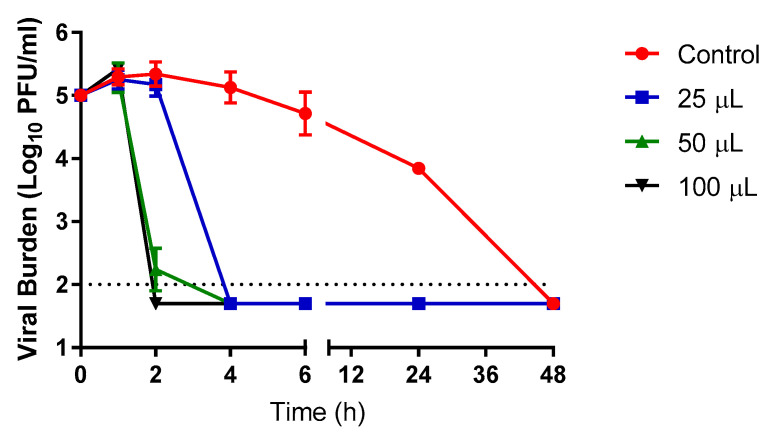
The effect of various volumes of liquid Propylamylatin formula on the ability of vapors to inactivate infectious SARS-CoV-2. A suspension of 10^5^ PFU of SARS-CoV-2 was exposed to vapors resulting from 25, 50, or 100 µL aliquots of liquid Propylamylatin formula and incubated at 37 °C. At various times post-Propylamylatin-formula vapor exposure, viral suspensions were harvested, and viral burden was quantified by plaque assay in Vero E6 cells. Viral burden is reported as log_10_ PFU/mL. Data points correspond to the geometric mean of two independent samples and error bars represent one standard deviation. The horizontal dashed line signifies the plaque assay limit of detection.

Experiments were also conducted to evaluate the influence of the Propylamylatin formula volume on the ability and timing of the vapors to inactivate SARS-CoV-2 at 37 °C. Vapors stemming from all starting liquid volumes of the Propylamylatin formula demonstrated potent anti-SARS-CoV-2 activity, driving the viral burden to levels below the assay limit of detection (Figure 3). However, the duration of time required to achieve complete inactivation of infectious SARS-CoV-2 was directly related to the liquid volume of the Propylamylatin formula at the start of the experiment (Figure 3). Larger volumes of Propylamylatin formula decreased viral loads faster than did smaller volumes of the formula. This was most evident at the 2 h post-exposure time point, as experimental arms with 25 µL of the Propylamylatin formula exhibited viral titers that were similar to the control (5.2-log_10_ PFU/mL) whereas arms with 50 and 100 µls of liquid had viral titers that were near (2.2-log_10_ PFU/mL) or below the plaque assay limit of detection, respectively (Figure 3). The volume of the Propylamylatin formula that yields an effective concentration of vapors to reduce the SARS-CoV-2 burden by 90% (EC_90_) was 53.44 µls at the 2 h time point. Infectious SARS-CoV-2 was not detected in any of the Propylamylatin formula experimental arms by 4 h post-exposure.

### 3.2. The Amount of Dissolved Propionic Acid and Isoamyl Hexanoates in Media Exposed to the Propylamylatin Formula Vapors

We measured the amounts of dissolved propionic acid and isoamyl hexanoates in tissue culture media (absent of virus) that were exposed to vapors volatilizing from various volumes of Propylamylatin formula over time when incubated at 37 °C. These experiments were performed to identify an exposure–response relationship between the propionic acid and/or isoamyl hexanoates and antiviral effect. We focused our evaluations on the early time points, as the viral burden was below the limit of detection in all of the Propylamylatin formula vapor-treated arms after 4 h of exposure. As expected, higher levels of propionic acid were detected when larger volumes of the Propylamylatin formula were allowed to volatilize and levels increased over time (Table 1). The Propylamylatin formula volume-dependent response was observed only at 2 h post-vapor exposure due to the fact that the virus was undetectable at all subsequent time points with the exception of the control (Figure 3). At 2 h, 2.6 µls of dissolved propionic acid was detected in the 25 µL experimental arm whereas 6.1–7.7 µls and 9.7–22.5 µls (depending on the quantification method used) was detected in the 50 and 100 µL experimental arms, respectively. By 4 h post-vapor exposure (when all experimental arms had undetectable viral titers), propionic acid levels were between 5.5 and 6.4 µls when 25 µls of the Propylamylatin formula was volatilized, 16.2 µL when 50 µL volatilized, and 20.5 µls when 100 µls was volatilized. Isoamyl hexanoates were not detected in the medium that was incubated in the presence of 25 or 50 µls of evaporated the Propylamylatin formula and existed at low levels (1.4 µls) when 100 µls was allowed to evaporate (Table 1).

### 3.3. Antiviral Activity of the Propylamylatin Formula against SARS-CoV-2 When Administered Directly into Viral Supernatants

**Figure 4:**
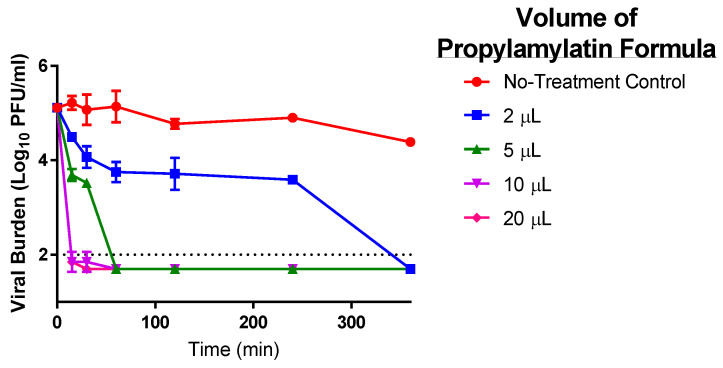
The antiviral effect of various volumes of the Propylamylatin formula to inactivate infectious SARS-CoV-2. A 1 mL suspension of 10^5^ PFU of SARS-CoV-2 was directly exposed to various volumes of liquid Propylamylatin formula and incubated at 37 °C. At various times post-Propylamylatin-formula exposure, viral suspensions were harvested, and the viral burden was quantified by plaque assay in Vero E6 cells. Viral burden is reported as log_10_ PFU/mL. Data points correspond to the geometric mean of two independent samples and error bars represent one standard deviation. The horizontal dashed line signifies the plaque assay limit of detection.

The Propylamylatin formula completely inactivated infectious SARS-CoV-2 virus when directly added to viral suspensions; however, the rapidity of inactivation was heavily influenced by the amount of formula administered (Figure 4). The addition of 10 µls (104.4 µmoles propionic acid with 10.2 µmoles of the isoamyl hexanoates) and 20 µls (208.8 µmoles propionic acid with 20.4 µmoles of the isoamyl hexanoates) of the Propylamylatin formula resulted in nearly undetectable levels of infectious virus after just 15 min of exposure, whereas the viral burden was equivalent to 3.7- and 4.5-log_10_ PFU/mL after the addition of 5 µL (52.2 µmoles propionic acid with 5.1 µmoles of the isoamyl hexanoates) and 2 µls (20.8 µmoles propionic acid with 2.0 µmoles of the isoamyl hexanoates), respectively, at the same time point. Viral burden was below the assay limit of detection at 60 min following the addition of 5 µls of the Propylamylatin formula and at 360 min (6 h) after adding 2 µls of agent. SARS-CoV-2 infectivity was stable throughout the experiment with viral titers ranging from ~5-log_10_ PFU/mL to 4.5-log_10_ PFU/mL over the 6 h study, suggesting that any loss of viral infectivity is due to the Propylamylatin formula and not natural degradation.

The EC_90_ volume of the Propylamylatin formula was calculated at individual time points as well as over the duration of the entire experiment using Hill models. The EC_90_ estimates decreased as exposure time increased, resulting in a value of 27.73 µls at 15 min, 9.30 µls at 30 min, and ~2.16 µls at 60 min to 240 min. The EC_90_ value for the entire 360 min study was determined to be 4.28 µls. These findings demonstrate the influence of exposure time on the antiviral activity of the Propylamylatin formula as well as the potency of this agent to inactivate infectious SARS-CoV-2.

### 3.4. The Effect of the Propylamylatin Formula on the pH of Viral Culture Media

The addition of the Propylamylatin formula to the viral diluent medium resulted in a clear color change of red to yellow, signifying a change in pH, and this change was dependent on the volume of agent added. We characterized the influence of the Propylamylatin formula over time on the pH of the medium used to dilute the virus in the direct injection studies. The pH decreased with increasing volumes of Propylamylatin formula, with a final value of 8.5 in the control, 6.0 in the 2 µL arm, 5.0 in the 5 µL arm, and 4.0 in the 10 µL and 20 µL arms (Table 2). The pH values were stable in all arms over the 24 h experiment, with the exception of the 10 µL experimental arm which started at a pH equivalent to 4.5 at 15 min post-exposure before finally reaching a stabilized (maximal) value of 4.0 after 30 min (Table 2). These findings indicate that the Propylamylatin formula creates an acidic environment in the medium upon addition.

### 3.5. Antiviral Activity of the Individual Components of the Propylamylatin Formula against SARS-CoV-2 When Administered Directly into Viral Supernatants

**Figure 5:**
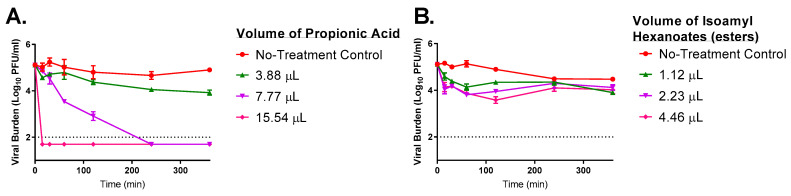
The antiviral effect of various volumes of the individual components of the Propylamylatin formula, propionic acid (**A**) and isoamyl hexanoates (**B**). The volumes evaluated correspond to the amount of that component present in a 5, 10, or 20 µL volume of Propylamylatin™, as acid to esters exist in a ratio 7:2 (*v/v*) in the Propylamylatin formula combination. A 1 mL suspension of 10^5^ PFU of SARS-CoV-2 was directly exposed to various volumes of propionic acid or isoamyl hexanoates and incubated at 37 °C. At various times post-exposure, viral suspensions were harvested, and viral burden was quantified by plaque assay in Vero E6 cells. Viral burden is reported as log_10_ PFU/mL. Data points correspond to the geometric mean of two independent samples and error bars represent one standard deviation. The horizontal dashed line signifies the plaque assay limit of detection.

The antiviral activity of each of the components that make up the Propylamylatin formula was evaluated against SARS-CoV-2 individually to determine the contribution of the antiviral effect of each component. Propionic acid and isoamyl hexanoates (esters) were examined as monotherapy at the proportions in which they exist in the formula, which is equivalent to a 7:2 (*v/v*) ratio of propionic acid to esters. For example, a 20 µL volume of the Propylamylatin formula consists of 15.54 µls of propionic acid and 4.46 µls of esters whereas a 5 µL volume of the Propylamylatin formula is comprised of 3.88 µls and 1.12 µls of propionic acid and esters, respectively. The 2 µL volume of Propylamylatin was not evaluated as an individual component because this would have required the addition of ~0.45 µls of esters, which is a volume that we could not accurately pipette. Propionic acid alone inhibited SARS-CoV-2 in an exposure-dependent manner, with larger volumes of acid providing a greater and more rapid decline in infectious viral burden (Figure 5A). The largest volume evaluated, 15.54 µls of propionic acid, resulted in an undetectable viral burden 15 min after exposure. The 7.77 µL volume provided a steady decline in infectious SARS-CoV-2, yielding undetectable levels of virus after 240 min (4 h) of exposure (Figure 5A). The smallest volume provided markedly less inhibition resulting in a decline of 0.98-log_10_ PFU/mL with 3.88 µls after 360 min post-addition. The EC_90_ value over the entire study is 11.50 µls for propionic acid.

The isoamyl hexanoates alone were substantially less effective at suppressing SARS-CoV-2 replication and the degree of inhibition was similar between all treatment arms, indicating the lack of an exposure–response relationship (Figure 5B). Thus, an EC_90_ value was not calculated for this data set. The majority of inhibition occurred during the first 1 h of exposure to the esters, as viral titers were relatively unchanged at the subsequent time points. These findings suggest that the propionic acid is the more active component in the Propylamylatin formula. But the addition of the esters to the propionic acid is necessary as the combination of these two components (as the Propylamylatin formula) provides substantially greater viral inhibition than either component individually (Figure 4 and Figure 5). This is particularly evident at the lower Propylamylatin formula volume (5 µls) which completely suppressed infectious SARS-CoV-2 (Figure 4), but the individual components of 3.88 µls of propionic acid and 1.12 µls of isoamyl hexanoates resulted in a minimal reduction of the viral burden (Figure 5).

### 3.6. The Effect on Antiviral Activity When Propionic Acid Is Substituted with Isobutyric Acid in Combination with Isoamyl Hexanoates (Esters)

**Figure 6:**
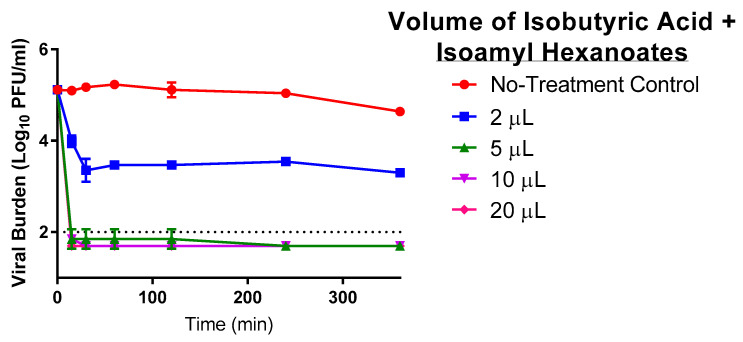
The antiviral effect of various volumes of isobutyric acid in combination with isoamyl hexanoates (esters) (at a ratio of 7:2, acid to esters) to inactivate infectious SARS-CoV-2. A 1 mL suspension of 10^5^ PFU of SARS-CoV-2 was directly exposed to various volumes of liquid the Propylamylatin formula and incubated at 37 °C. At various times post-exposure, viral suspensions were harvested, and viral burden was quantified by plaque assay in Vero E6 cells. Viral burden is reported as log_10_ PFU/mL. Data points correspond to the geometric mean of two independent samples and error bars represent one standard deviation. The horizontal dashed line signifies the plaque assay limit of detection.

We evaluated the anti-SARS-CoV-2 activity of isobutyric acid, the original product produced by the Muscodor sp., in combination with the isoamyl hexanoates that comprise the Propylamylatin formula to determine how the substitution of isobutyric acid with propionic acid influences the antiviral effect of the Propylamylatin formula via direct injection assays. The same ratio of 7:2 acid to esters was utilized for these studies. Similar to the Propylamylatin formula, isobutyric acid in combination with the isoamyl hexanoantes rapidly inactivated infectious SARS-CoV-2 (Figure 6). The isobutyric acid + ester mixture at volumes of 5, 10, and 20 µls yielded viral titers at or below the assay limit of detection after only 15 min of exposure. In the 2 µL arm, viral titers were reduced 10-fold yielding a viral burden of 4.0 log_10_-PFU/mL at the same time point. Infectious virus further declined to ~3.5-log_10_ PFU/mL by 30 min post-administration and titers remained at this level for the duration of the experiment (Figure 6). These findings demonstrate that the inactivation of SARS-CoV-2 by isobutyric acid in combination with the esters is heavily influenced by volume and exposure time, as also described for the Propylamylatin formula (Figure 4). The degree of viral inhibition is very similar between the isobutyric acid/ester combination and the propionic acid/ester combination (Propylamylatin formula). The overall EC_90_ volume for the isobutyric acid + ester mixture was 2.58 µls, which is comparable to the 4.28 µls reported for that of the Propylamylatin formula.

We also measured the effect of the isobutyric acid and esters in combination with the pH of the viral diluent media. The pH levels for this combination were identical to those reported for the Propylamylatin formula over time (Table 2). These findings indicate that the isobutyric acid and esters in combination create an acidic environment that is identical to the Propylamylatin formula upon addition to the medium used to dilute SARS-CoV-2. Additionally, we have studied the effects of acetic acid alone in this assay system. Acetic acid is more acidic than propionic acid with pKa’s of 3.75 and 4.87, respectively. Acetic acid alone was not as potent against SARS-CoV-2 when compared to propionic acid alone, as acetic acid resulted in an EC_90_ value of 19.42 µls whereas the EC_90_ was 11.13 µls for propionic acid. These results suggest that acidity is not the only mechanism of action for virus inactivation and that other structural features of the propionic and isobutyric acids can be attributed to antiviral activity.

## 4. Discussion

The COVID-19 pandemic continues to threaten global public health, spreading readily from person to person and resulting in significant illness and an unacceptably high number of deaths. Although numerous preclinical and clinical investigations have been conducted to identify effective drug candidates for the treatment and prevention of COVID-19, there are currently no antiviral agents approved for this serious disease. Natural products derived from plant, fungi, and marine sources have been, and are still, frequently used to treat viral infections, particularly those that cause respiratory illnesses such as rhinovirus and influenza virus [2,5,7,8,9,10,11]. Natural products are attractive therapies for viral as well as other microbial infections because they are often accessible in nature (making them low in cost), amenable to mass production, and have generally good safety profiles [9]. Moreover, the lack of available antiviral drugs for many ubiquitous viral diseases makes natural-based remedies a logical source of treatment for these infections, especially in developing countries [12,13]. For these studies, we aimed to evaluate the Propylamylatin formula against SARS-CoV-2, in an attempt to identify a potentially effective treatment strategy to combat COVID-19.

Because the antimicrobial activity of the natural fungus-derived VOCs was originally observed in the gas phase, we first evaluated the Propylamylatin formula in the gaseous state against SARS-CoV-2 at room temperature (18 °C) and body temperature (37 °C). This initial study was conducted in order to determine if the Propylamylatin formula might possess any antiviral activity and if so, at what concentrations. Ultimately, this information would serve as a guide for more comprehensive and better-controlled studies using a direct injection method of the Propylamylatin formula into viral suspension. As previously described by others, infectious SARS-CoV-2 virus is naturally more stable at cooler temperatures with the viral burden persisting longer at room temperature compared to body temperature [14,15]. The Propylamylatin formula vapors were effective against SARS-CoV-2 at both temperatures, resulting in undetectable levels of virus after exposure. However, viral inactivation was substantially faster at higher temperatures. This is likely due to the fact that the Propylamylatin formula volatizes more rapidly at higher temperatures. The impressive ability of the formula vapors to inactivate high levels (~10^5^ PFU/mL) of SARS-CoV-2 at room temperature has important implications, as this agent may serve as a way to inactivate the virus on different surfaces including instruments and personal protective equipment. Studies evaluating the disinfecting potential of the Propylamylatin formula vapors against SARS-CoV-2 on surfaces made of plastic, glass, metal, etc. are currently ongoing.

Measuring the levels of dissolved propionic acid and isoamyl hexanoates in the tissue culture medium post-exposure to the vapors volatizing from the Propylamylatin formula at 37 °C revealed that propionic acid was readily detected but the esters (isoamyl hexanoates) were only present (at very low levels) when high starting volumes of compound were employed. These findings indicate that the majority of anti-SARS-CoV-2 activity was likely due to the propionic acid under this route of administration.

Per our hypothesis, our initial interest in the Propylamylatin formula was as a potential antiviral strategy against the virus causing COVID-19; thus, we conducted direct injection experiments where the Propylamylatin formula was directly added to a SARS-CoV-2 suspension at 37 °C to simulate body temperature. The addition of at least 10 µls of the Propylamylatin formula into 1 mL suspension of 10^5^ PFU of SARS-CoV-2 completely inactivated SARS-CoV-2 to levels below the limit of detection after only 15 min of exposure. Assessing the individual components of the Propylamylatin formula revealed that indeed, as identified from the gas phase experiments, the propionic acid provided the majority of antiviral effect compared to the isoamyl hexanoates. However, the degree of inhibition was markedly greater when the propionic acid and the isoamyl hexanoates were combined, indicating synergy. These findings for antiviral activity are in concordance with what has been reported for the antimicrobial activity of the Propylamylatin formula against bacteria and fungi [2,3,4,5].

Isobutyric acid is the small weight molecular acid identified in the original VOCs made by the fungus. Isobutyric acid in combination with the isoamyl hexanoates was slightly more potent overall than the Propylamylatin formula (which contains propionic acid with the isoamyl hexanoates) yielding EC_90_ values of 2.58 µls and 4.28 µls, respectively. This difference in EC_90_ is mainly attributed to the 5 µL volume of the isobutyric mixture which completely inhibited SARS-CoV-2 after 15 min of exposure, yielding inhibitory kinetics that were identical to the 10 µL and 20 µls volumes. A 5 µL volume of the Propylamylatin formula provided complete inhibition after 60 min of exposure. These differences in antiviral activity are relatively negligible. Moreover, there are several benefits to using propionic acid over isobutyric acid, the most obvious being the unattractive odor of isobutyric acid, which smells like rancid butter. Propionic acid also has an unpleasant odor, but it is less pungent than that of isobutyric acid.

The degree and rapidity of viral inactivation via the Propylamylatin formula is impressive. It is hypothesized that the agent may inactivate the virus via interaction with the receptor binding domain (RBD) of the SARS-CoV-2 spike protein, which plays a critical role in viral attachment to the cellular angiotensin-converting enzyme 2 (ACE2) receptor [16]. A polybasic cleavage site that is 10 nm from the RBD has been identified on the spike protein and shown to enhance the binding affinity of RBD to ACE2 [17]. Blocking this positively charged site (arginine) with negatively charged anions in the presence of a strong lipophilic agent should effectively neutralize this critical site and render it ineffective. This phenomenon was shown to be true by Qiao et al., as the addition of a synthetic peptide GluGluLeuGlu to SARS-CoV-2 reduced the binding affinity of RBD to ACE2 by 34% [17]. The reduction of binding strength would feasibly reduce the infectivity of the virus. Thus, this may be one mechanism by which the Propylamylatin formula inactivates SARS-CoV-2. A second mechanism by which this agent may act is through its ability to create an acidic environment. The pH was inversely proportional to antiviral activity, with lower pH values resulting in a more rapid decline in viral burden. Others have reported that the infectivity of coronaviruses including SARS-CoV (causative agent of the 2002 to 2003 SARS outbreak) are very sensitive to pH extremes, as lower pH values were able to completely inactivate SARS-CoV [18]. Thus, the Propylamylatin formula may rapidly inactivate infectious SARS-CoV-2 by blocking the polybasic cleavage site on the spike protein and/or creating an acidic environment that renders the virus non-infectious.

Our studies illustrate the therapeutic potential of the Propylamylatin formula for the treatment of COVID-19. As shown above, a volume of only 10 µls was required to completely inactivate infectious SARS-CoV-2 after only 15 min of contact time. This amount of formula is equivalent to 1% of the total volume of liquid in the suspension (10 µls of the Propylamylatin formula into 1000 µls of virus suspension) and represents the exact amount of the Propylamylatin formula that was present in the electrolyte solution originally administered to piglets suffering with PEDv. These findings warrant further investigations to determine if the level of the Propylamylatin formula required to inactivate SARS-CoV-2 is physiologically achievable in humans and whether it will have a direct application to treat human COVID-19.

Gastrointestinal symptoms have been reported in patients with COVID-19 [19,20] and SARS-CoV-2 has been shown to infect human gut enterocytes [21]. Therefore, a 1% solution of Propylamylatin formula may be sufficient to inhibit viral replication in the digestive system, thereby alleviating gastrointestinal symptoms. Respiratory symptoms may also be relieved with the formula. The Propylamylatin formula could possibly be applied as a topical agent in the nasal cavities to inactivate infectious virus in the nose, which is the initial site of infection. It has been proposed that aspiration from the nasal cavity into the oral cavity may be a route for the virus to transverse from the upper respiratory system into the lower respiratory system, resulting in more severe disease outcomes [22,23]. Thus, a 1% solution of the Propylamylatin formula as a mouthwash may inhibit any virus in the oral cavity from reaching the lungs and preventing pneumonia as well as other more severe disease outcomes. Finally, Propylamylatin formula when administered via an atomizer, nebulizer, or intubation directly into lung tissue may be helpful in reducing viral load in these tissues. Additional studies are required to address these hypotheses.

There are some weaknesses associated with our study. First, our experiments were conducted with a single strain of SARS-CoV-2. Studies will be repeated with new viral variants, including the mutated United Kingdom and South African strains, to ensure that the Propylamylatin formula is effective against a variety of SARS-CoV-2 strains. Secondly, we plan to evaluate toxicities associated with the 1% solution in the lung tissue using small rodent models.

In conclusion, we have demonstrated that the Propylamylatin formula holds promise as a therapeutic strategy to be further investigated for patients afflicted with COVID-19, a disease for which there are no approved antiviral therapies. The potent direct inhibition of infectious SARS-CoV-2 suggest that further preclinical (in vitro and in vivo) studies are warranted for this virus and other coronaviruses.

## Figures and Tables

**Table 1:** The amount of dissolved propionic acid and isoamyl hexanoates in tissue culture medium incubated in the presence of volatilized Propylamylatin formula resulting from different liquid volumes at 37 °C.

Amount of Propylamylatin Formula (µls)	Time Post-Exposure to Propylamylatin Formula Vapors (h)	Amount of Propionic Acid via Titration with 1 M NaOH (µls)	Amount of Propionic acid via GC/FID ^a^ (µls)	Amount of Isoamyl Hexanoates via GC/MS ^b^ (µls)
25	2	2.6	ND ^c^	ND ^c^
	4 ^d^	5.5	6.4	0
	6	8.6	ND ^c^	ND ^c^
50	2	7.7	6.1	0
	4 ^d^	16.2	ND ^c^	0
	6	22	ND ^c^	0
100	2 ^d^	9.7	22.5	1.4
	4	20.5	ND ^c^	ND ^c^
	6	25.5	ND ^c^	ND ^c^

^a^ Gas chromatography/ flame ionization detection (GC/FID); ^b^ gas chromatography/ mass spectroscopy (GC/MS); ^c^ ND: not determined; ^d^ The time point in which viral burden was below the assay limit of detection.

**Table 2:** The pH of tissue culture medium used to dilute SARS-CoV-2 at various times post-exposure to various volumes of Propylamylatin formula.

Amount of Propylamylatin Formula (µL)	Time Post-Exposure to the Propylamylatin Formula (mins)						
	15	30	60	120	240	360	1440 (24 h)
0	8.5	8.5	8.5	8.5	8.5	8.5	8.5
2	6.0	6.0	6.0	6.0	6.0	6.0	6.0
5	5.0	5.0	5.0	5.0	5.0	5.0	5.0
10	4.5	4.0	4.0	4.0	4.0	4.0	4.0
20	4.0	4.0	4.0	4.0	4.0	4.0	4.0

## Data Availability

The data presented in this study are available on request from the corresponding author.
